# Orphan drug development in alpha-1 antitypsin deficiency

**DOI:** 10.1038/s41598-022-19707-2

**Published:** 2022-09-15

**Authors:** Franziska C. Trudzinski, Maria Ada Presotto, Emanuel Buck, Felix J. F. Herth, Markus Ries

**Affiliations:** 1 Department of Pneumology and Critical Care Medicine, Thoraxklinik, Translational Lung Research Center Heidelberg (TLRC-H), Member of the German Center for Lung Research (DZL), University of Heidelberg, Röntgenstraße 2, 69126 Heidelberg, Germany; 2 Pediatric Neurology and Metabolic Medicine, Center for Pediatrics and Adolescent Medicine, University Hospital Heidelberg, Heidelberg, Germany; 3 Center for Virtual Patients, Faculty of Medicine, University of Heidelberg, Heidelberg, Germany; 4 Center for Rare Diseases, University Hospital Heidelberg, Heidelberg, Germany

**Keywords:** Medical research, Drug development

## Abstract

Alpha-1 antitrypsin deficiency (AATD, OMIM #613490) is a rare metabolic disorder affecting lungs and liver. The purpose of this study is to assess the impact of the US orphan drug act on AATD by providing a quantitative clinical-regulatory insight into the status of FDA orphan drug approvals and designations for compounds intended to treat AATD. This is across-sectional analysis of the FDA database for orphan drug designations. Primary endpoint: orphan drug approvals. Secondary endpoint: orphan drug designations by the FDA. Close of database was 16 July 2021. STROBE criteria were respected. Primary outcome: one compound, alpha-1-proteinase inhibitor (human) was approved as an orphan drug in 1987 with market exclusivity until 1994. Secondary outcome: sixteen compounds received FDA orphan drug designation including protein, anti-inflammatory, mucolytic, gene, or cell therapy. Drug development activities in AATD were comparable to other rare conditions and led to the FDA-approval of one compound, based on a relatively simple technological platform. The current unmet medical need to be addressed are extrapulmonary manifestations, in this case the AATD-associated liver disease. Orphan drug development is actually focusing on (1) diversified recombinant AAT production platforms, and (2) innovative gene therapies, which may encompass a more holistic therapeutic approach.

## Introduction

Alpha-1 antitrypsin deficiency (OMIM #613490) is a rare metabolic disorder caused by mutations in the SERPINA1 gene^1,2^. The estimated prevalence based on European data is 20/100,000^3^. The disease is inherited in an autosomal codominant manner. In addition to the deficiency mutant protease inhibitor (Pi) Z, which results from the substitution of a single amino acid, Glu342Lys responsible for the majority of clinically apparent cases, several genetic variants have been described that can be associated with severe AATD deficiency^4–8^. Alpha-1 antitrypsin (AAT) is mainly synthesized in the endoplasmic reticulum of the liver^2^. PiZ allele carriers produce a misfolded but basically functional protein, that tend to form polymers which accumulate in the hepatocytes resulting in only a small amount of protein being secreted. AAT is an antiprotease that protects lung tissue from proteolytic damage by inactivating neutrophil elastase (NE) released by neutrophil granulocytes in response to respiratory infections. Due to their antiprotease deficiency, homozygous PiZ allele carriers show accelerated loss of lung tissue, particularly in association with smoking^1^. The protein has also several immunomodulatory properties^9^. AATD is associated with different autoimmune diseases^10,11^ and other pulmonary conditions such as bronchiectasis, caused by the inflammatory burden of overwhelming NE^12^. The accumulation of AAT polymers in the liver via proteotoxic stress can lead to severe hepatopathy and hepatocellular carcinoma^13,14^. Patients with AATD usually present with respiratory symptoms mainly cough and dyspnoea due to the progressive development of emphysema, often interpreted as non-hereditary emphysema, so that several years usually elapse between symptom onset and diagnosis^2^.

There is a great need for easy-to-use or sustainable treatment options, possibly tailored to the individual disease phenotype that address extrapulmonary manifestations especially AATD liver disease in addition to lung disease. Therefore, the orphan drug research pipeline for AATD is of interest.

The U.S. Orphan Drug Act passed in 1983 and provides various incentives to pharmaceutical companies with the intention to stimulate drug development for the treatment of rare diseases. These incentives include a seven years' marketing exclusivity, tax credit for 50% of clinical trial costs, protocol assistance, Food and Drug Administration fee waiver, and orphan products grants program^15^. By 16 July 2021 (close of database for this analysis), a total of 998 orphan drugs were approved by the FDA^16^. Similar orphan drug policies were subsequently introduced in other jurisdictions worldwide, e.g., Singapore 1991, Japan 1993, Australia 1998, the European Union 2000^17,18^.

Because the U.S. orphan drug legislation is the oldest worldwide, and the United States is an important market for the pharmaceutical industry, the purpose of this study is to assess the impact of the U.S. orphan drug act on AATD by providing a quantitative clinical-regulatory insight into the status of FDA orphan drug approvals and designations for compounds intended to treat AATD. This analysis gives an insight into dynamics, productivity, innovation, timelines, opportunities and limitations of orphan drug development for AATD from an FDA-centered perspective. It provides transparency, raises awareness of gaps, and has the potential to inspire further research and development activities worldwide.

## Methods

This is a cross-sectional observational study. Strengthening the Reporting of Observational Studies in Epidemiology (STROBE) criteria were respected in design, execution, analysis, and reporting^19^.

### Endpoints

The primary endpoint of this analysis was numbers and nature of orphan drug approvals by the FDA intended to treat AATD. The secondary endpoint was numbers and nature of orphan drug designations by the FDA intended to treat AATD.

### Data sources

In order to search for orphan drug designations and approvals, accessed the FDA Orphan Drug Product designation database at https://www.accessdata.fda.gov/scripts/opdlisting/oopd/ on 16 July 2021. Search terms for the field "orphan designation" was "antitrypsin" and "proteinase". This search delivered 14 results and two results, respectively, all specific to alpha-1-antitrypsin deficiency. The results were downloaded in excel format. The following available variables were analysed: generic name of compound, year of FDA orphan drug designation, orphan designation (intended treatment indication), orphan designation status (designated, withdrawn, or approved), name and country of sponsor company.

In addition, we verified whether there were approved drugs for the treatment of alpha-1-antitrypsin deficiency, that were not listed in the U.S. Food and Drug Administration Orphan Drug Product database. We therefore performed a full text search in the FDA drug label database^20^. Search terms were “antitrypsin” and “proteinase” in the section “indications and usage”. Identified drugs from the search in FDALabel were juxtaposed to the approved compounds identified from the search in Orphan Drug Product designation database^21^. The search term “antitrypsin” delivered four, the search term “proteinase” one result. In addition, we cross-validated whether or not the compounds identified from the search in FDALabel were registered as orphan drugs in the U.S. Food and Drug Administration Orphan Drug Product database^21^.

### Definitions

Compounds were classified into functionally meaningful types based on their biochemical properties, molecular mechanisms of action, or therapy platforms^22^. These types included: protein therapy, anti-inflammatory, mucolytic, gene therapy, and cell therapy.

### Statistical analysis

Standard techniques of descriptive statistics were applied. Analysed variables were: generic name, classification, year of orphan drug designation, orphan designation (i.e., intended therapeutic indication), orphan designation status (designated, withdrawn, approved), FDA orphan approval status, approved label indication, year of marketing approval, year of exclusivity end date, sponsor company and country^21,22^.

Missing data were not imputed.

## Results

### Primary endpoint: orphan drug approvals by the FDA

One compound, alpha-1-proteinase inhibitor (human) was approved as an orphan drug in 1987 with market exclusivity until 1994, after having received orphan drug designation in 1984.

### Secondary endpoint: FDA orphan drug designations

**Figure 1:**
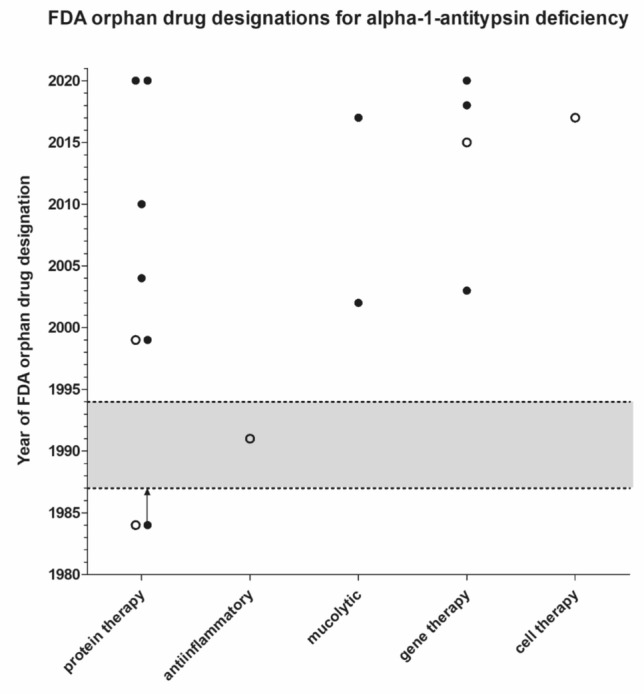
Type of compound intended to treat alpha-1 antitrypsin deficiency by year of FDA orphan drug designation. Full circles: designated compound. Open circles: withdrawn orphan drug designation. Alpha1-proteinase inhibitor (human) received orphan drug designation in 1984 and was approved by the FDA in 1987, the arrow indicates the development time between orphan drug designation and FDA orphan drug approval. The grey area shows the period of marketing exclusivity for this compound between 1987 and 1994.

Between 1984 and 16 July 2021 (close of database), sixteen compounds intended to treat alpha-1 antitypsin deficiency received FDA orphan drug designation (Fig. 1). Five compounds were subsequently withdrawn (Table 1). Of interest, another alpha1-proteinase inhibitor (human) received orphan drug designation in 1999 which was subsequently withdrawn and not approved under the U.S. orphan drug legislation, whereas alpha-1-proteinase inhibitor human sponsored by the same manufacturer was granted FDA approval in 2003 (Table 2).

**Table 1:** FDA orphan drug designations for compounds intended to treat alpha-1-antitypsin deficiency in reverse chronological order sorted by year of orphan drug designation.

Generic name of compound	Classification	Year of FDA orphan drug designation	Orphan designation	Orphan designation status	Sponsor company	Country of sponsor company
CHO cell line produced human alpha-1 antitrypsin (CHO-AAT) protein	Protein therapy	2020	treatment of alpha-1 antitrypsin deficiency	Designated	Caravella Biopharma SA	Switzerland
A synthetic double-stranded RNA oligonucleotide conjugated to N-acetyl-D-galactosamine aminosugar residues	Gene therapy	2020	Treatment of alpha-1 antitrypsin deficiency	Designated	Dicerna Pharmaceuticals, Inc	United States
Recombinant human alpha-1 antitrypsin from Oryza sativa	Protein therapy	2020	Treatment of alpha-1 antitrypsin deficiency	Designated	Wuhan Healthgen Biotechnology Corporation	China
Double stranded oligomer ADS-001 RNA interference-based liver targeted therapeutic	Gene therapy	2018	Treatment of alpha-1 antitrypsin deficiency	Designated	Arrowhead Research Corporation	United States
Three-dimensional bioprinted therapeutic liver tissue	Cell therapy	2017	Treatment of alpha-1 antitrypsin deficiency	Designated/withdrawn	Organovo Inc	United States
Hyaluronic acid	Mucolytic	2017	Treatment of emphysema due to alpha1-antitrypsin deficiency	Designated	Gerard M. Turino, MD	United States
Double stranded oligomer AD00370 RNA interference-based liver targeted therapeutic	Gene therapy	2015	Treatment of Alpha-1 Antitrypsin deficiency	Designated/withdrawn	Arrowhead Research Corporation	United States
Alpha1 proteinase inhibitor (human)	Protein therapy	2010	Treatment of emphysema secondary to congenital alpha1-antitrypsin deficiency	Designated	Grifols Therapeutics, Inc	United States
Alpha1-Proteinase Inhibitor (Human)	Protein therapy	2004	Inhalation therapy for the treatment of congenital deficiency of alpha1-proteinase inhibitor	Designated	Kamada Ltd	Israel
Recombinant adeno-associated virus alpha 1-antitrypsin vector	Gene therapy	2003	Treatment of alpha1-antitrypsin deficiency	Designated	University of Massachusetts Medical School	United States
Hyaluronic acid	Mucolytic	2002	Treatment of emphysema in patients due to alpha-1 antitrypsin deficiency	Designated	CoTherix	United States
Alpha1-proteinase inhibitor (human)^b^	Protein therapy	1999	For slowing the progression of emphysema in alpha1-antitrypsin deficient patients	Designated/withdrawn	CSL Behring L.L.C	United States
Transgenic human alpha 1 antitrypsin	Protein therapy	1999	Treatment of emphysema secondary to alpha 1 antitrypsin deficiency	Designated	PPL Therapeutics (Scotland) Limited	United Kingdom
Recombinant secretory leucocyte protease inhibitor	Anti-inflammatory	1991	Treatment of congenital alpha-1 antitrypsin deficiency	Designated/withdrawn	Amgen Inc	United States
Alpha-1-antitrypsin (recombinant DNA Origin)	Protein therapy	1984	As supplementation therapy for alpha-1-antitrypsin deficiency in the ZZ phenotype population	Designated/withdrawn	Chiron Corporation	United States
Alpha1-proteinase inhibitor (human)^a^	Protein therapy	1984	For replacement therapy in the alpha-1-proteinase inhibitor congenital deficiency state	Designated/approved	Bayer Corporation	United States

Close of database: 16 July 2021.

^a^Approved by the FDA in 1987 “for chronic replacement therapy of individuals having congenital deficiency of alpha1- proteinase inhibitor with clinically demonstrable panacinar emphysema” with marketing exclusivity until 1994.

^b^Alpha-1-proteinase inhibitor human sponsored by the same manufacturer was approved by the FDA in 2003 (Table 2).

**Table 2:** FDA approved compounds for the treatment of alpha-1-antitypsin deficiency in reverse chronological order by year of FDA approval.

Generic name of compound	Initial FDA approval	Galenics	Reference (accessed 5 August 2021)
alpha.1-proteinase inhibitor human injection, solution	2010	Solution	https://dailymed.nlm.nih.gov/dailymed/getFile.cfm?setid=83473cbb-48e4-42a2-81b6-4c851423da7b&type=pdf
alpha-1-proteinase inhibitor human^a^	2003	Lyophilized powder	https://dailymed.nlm.nih.gov/dailymed/getFile.cfm?setid=0c3354b5-a1d8-4f98-ad55-2eafe4265c4e&type=pdf
alpha-1-proteinase inhibitor (human)	2002	Lyophilized powder	https://dailymed.nlm.nih.gov/dailymed/getFile.cfm?setid=a9a5b46e-04da-41bd-bb5f-c4936b664fef&type=pdf
alpha1-proteinase inhibitor (human) injection, solution (human)^b^	1987	Solution	https://dailymed.nlm.nih.gov/dailymed/getFile.cfm?setid=2b620e6b-6a24-0957-1288-ae529c4cc3a2&type=pdf
alpha-1-proteinase inhibitor (human)^b^	1987	Lyophilized powder	https://dailymed.nlm.nih.gov/dailymed/getFile.cfm?setid=91edab72-c889-470e-8315-1798b5548dca&type=pdf

Close of database 5 August 2021.

^a^Alpha1-proteinase inhibitor (human) sponsored by the same manufacturer received orphan drug designation which was subsequently withdrawn (Table 1).

^b^Both compounds were listed with very similar trade names in the FDA orphan drug designation database as alpha1-proteinase inhibitor (human), i.e. Prolastin C liquid, Prolastin C vs. Prolastin).

Protein therapies with orphan drug designations differed in pharmaceutical manufacturing methods, i.e. their origins were either human, recombinant DNA, transgenic, made in Oryza sativa (rice), or CHO cell lines (Table 1). Gene therapies with orphan drug designations included a synthetic double-stranded RNA oligonucleotide, double stranded oligomer ADS-001 RNA interference-based liver targeted therapeutic, and a recombinant adeno-associated virus alpha-1 antitrypsin vector. An orphan drug designation for a cell therapy (three-dimensional bioprinted therapeutic liver tissue) was granted in 2017, but withdrawn subsequently. The mucolytic hyaluronic acid received orphan drug designation in 2002 and 2017, respectively, but no FDA approval. The anti-inflammatory recombinant secretory leucocyte protease inhibitor received orphan drug designation in 1991 which was later withdrawn. Fourteen orphan drug designation sponsors were pharmaceutical companies, ranging from big pharma to smaller, specialized biopharmaceutical companies. One sponsor was an academic institution, and one sponsor an individual physician. Thirteen sponsors were based in the United states, and one, respectively, in China, Israel, Switzerland, and the United Kingdom.

Currently, i.e. by 5 August 2021, there are five versions of alpha-1-proteinase inhibitor drug products approved by the FDA. They differ in galenic formulation, three of them are delivered as lyophilized powder and two as a solution (Table 2). Three alpha-1-proteinase inhibitor drug products were approved by the FDA after the end of market exclusivity of the original alpha-1-proteinase inhibitor in 1994 (Table 2).

## Discussion

The objective of this study was to analyse the FDA approvals and orphan drug designations for compounds to treat alpha-1 antitypsin deficiency (AATD) in order to gain a comprehensive overview of the status of current therapy developments for this rare hereditary disease.

### Primary endpoint: orphan drug approvals by the FDA

Since 1984, only one compound out of 16 orphan drug designations (= 6%) currently received FDA approval for the treatment of alpha-1-antitrypsin deficiency. In general, drug development in orphan indications appear to have a higher success rate than non-orphan indications. In a recent report from 2021, the likelihood of success from phase I to approval was 5.9% for chronic high prevalence diseases compared with a success rate of 17% for rare diseases^23^. As drug development in AATD currently is ongoing, it is possible that the current success rate of 6% increases if the drug development programs prove to be successful. A few non-orphan drug licenses listed in Table 2 appear to reflect a commercial strategy where the company may have felt that the orphan drug legislation did not offer additional advantages. This phenomenon was rather an exception than the rule in the overall AATD drug development portfolio which heavily relied on the orphan drug development pathway. The precise reasons for these choices remain, unfortunately, unknown. We speculate that, in general, the non-orphan regulatory drug development approach in rare diseases is chosen only in exceptional, infrequent circumstances, because of the various tangible incentives and benefits that the U.S. orphan drug act provides to the pharmaceutical industry.

Administration of purified human AAT is the only specific treatment; prospective randomized-controlled and open-label studies have shown a slowed decline in lung density with weekly infusions of 60 mg AAT/kg^24,25^. Whether AAT augmentation therapy has a positive effect on the extrapulmonary manifestations of the disease is currently unproven; moreover, weekly i.v. therapy is time-consuming and the treatment costs are high. In addition to preventive measures such as abstinence from nicotine, alcohol and the prompt treatment of respiratory infections, various symptomatic therapies for emphysema e.g. bronchodilators, inhaled steroids, the administration of oxygen or non-invasive ventilation are available if needed^26^.

Of note, alpha-1-proteinase inhibitors of human origin are subjected to availability of plasma donors and, despite manufacturing precautions, the risks related to plasma donations, e.g., virus contamination or Creutzfeldt-Jakob disease. Interestingly, the development of a recombinant alpha-1-proteinase inhibitor, which would mitigate these risks, has never been successful until close of database. One example for successful transition from donor-based protein sources to recombinant production is Gaucher disease, a rare neurogenetic disorder due to the inherited deficiency of the enzyme glucocerebrosidase. Here, the missing enzyme was initially produced from collected placenta tissue (alglucerase, approved by the FDA in 1994) and subsequently switched swiftly towards recombinant production in Chinese hamster ovary cell cultures (imiglucerase, approved by the FDA in 1994)^22,27^.

Early identification of gene carriers and education about necessary preventive measures such as nicotine abstinence and vaccination are essential for all individuals with severe AAT deficiency (genotype PI ZZ, ZNull or NullNull and other rarer gene variants) to avoid premature loss of lung function^28^. AAT augmentation therapy, as the only available therapeutic option with a causal approach, is usually administered in the late stages of the disease and therefore does not contribute to the prevention of lung disease, but only serves to slow the progression of the disease. An important characteristic of the disease that can be insufficiently addressed by early detection and appropriate lifestyle changes such as alcohol abstinence and weight reduction is AATD liver disease. Approximately one third of adults with PI ZZ AAT deficiency develop clinically significant liver fibrosis during the course of the disease^29,30^. Despite more than three decades of intensive research activity, no drug has yet been approved for this manifestation of AATD.

Major limitations of alpha-1-proteinase inhibitor augmentation therapy are its inconvenient application and lack of effect on extrapulmonary manifestations, which defines the current unmet medical need. The attempt of alternative alpha-1-proteinase inhibitor applications as well as the efforts to develop inhaled therapies^31^ have not yet led to a marketing approval. The possibility of intravenous self-administration at home allows suitable patients some freedom despite fixed weekly treatment schedules, at least because of the time savings and flexibility of self-administration^32^.

With respect to liver manifestation, emerging RNAi-based therapeutics targeting hepatic Z-AAT production currently in development may, if proven to be safe and efficacious, benefit patients with AATD-associated liver disease in the future^33,34^.

### Secondary endpoint: orphan drug designations by the FDA

Alpha-1-proteinase inhibitors of human origin were the first compounds intended to treat AATD that received an orphan drug designation in 1984 (Fig. 1).

Only one new compound (an anti-inflammatory) received an orphan drug designation during the marketing exclusivity period of the protein therapy alpha1-proteinase inhibitor (human) (Fig. 1), whereas thirteen orphan drug designation were granted after expiration of this marketing exclusivity in 1994. This could mean that exclusivity protected the pharmaceutical market whereas the absence of marketing exclusivity in a drug development environment with previously demonstrated success in orphan drug approval may stimulate innovation.

There are both commercial as well as economical aspects that play a role in drug development for rare diseases. From a patient’s perspective, the driver should be the unmet medical need. From a pharmaceutical company’s perspective, the main leitmotiv is returns on investment influenced by likelihood of success in the clinical development program and commercialization^27^. Therefore, as further explained in the introduction, in 1983, the US Orphan Drug Act passed, in order to provide incentives to pharmaceutical companies to invest into the development of drugs for rare diseases^15^. The output and impact can vary: medicines developed under the US orphan drug act in the very active field of rare cancers were in general very innovative, and had shifted from non-cytotoxic agents to targeted therapies^21,35^. Likewise, in lysosomal storage diseases, orphan drug development was very innovative with “a drug made for a disease”^22,27^. In lysosomal storage disorders, success factors for approval included disease prevalence, the choice of endpoints, regulatory precedence and technology platform^22,27^. In contrast to this, the development output in rare epilepsies and rare rheumatological diseases delivered, in general, little innovation (although there were notable exceptions such as stiripentol for Dravet’s syndrome), or focused mainly on compounds with other indications or similar molecular pathways, but further research and development activities are ongoing^36,37^. A better understanding of the genetics of epilepsy may improve pharmacological management and further inform and stimulate drug development in this field^38^. Overall, the drug development activities in AATD were quantitively similar to the spectrum observed within the family of lysosomal storage diseases, but the technological approach of the FDA-approved therapy for AATD remains rather simple and is still based on human protein sources. Of interest, four of 17 sponsoring institutions (24%) were based outside the U.S. in industrialized countries. Commercial sponsors ranges from big pharma to smaller, specialized biopharmaceutical companies^39^. The low number of orphan drug designations from academic institutions is not surprising, as there are no incentives specifically encouraging academics to seek orphan designation which is a well-known but neglected problem. Barriers may include inadequate structures, funding issues, career perspectives, and a lack of cross-functional capabilities (e.g., regulatory, intellectual property, clinical operations, translational medicine, or biostatistics support).

### Limitations and directions for future research

As pointed out previously, this analysis has some limitations that have to be taken into account for the correct interpretation of the present findings^21,22,27,35–37^. Guided by strategic and patent related considerations manufacturers may choose not to reveal the intent to develop a drug in a publicly visible way at an early stage which may have influenced the timeline to approval in Fig. 1. This analysis focusses intentionally on the FDA perspective^40^ and did therefore not consider regulatory jurisdictions elsewhere. Because in general drug development for rare diseases is a global endeavour, we regard the findings of the present analysis generalizable within the context of the limitations described^21^. This report serves as a baseline for future progress.

## Conclusion

Alpha-1 antitrypsin deficiency is a rare disease. Drug development activities in the field were comparable to other rare, treatable conditions (e.g., lysosomal storage diseases), and led to the FDA-approval of one compound, based on a relatively simple technological platform, meanwhile available in different galenic versions, which renders the condition treatable. However, alpha-1-proteinase inhibitor augmentation therapy is dependent on human donors and theoretically subjected to infectious risks. The current unmet medical need to be addressed are extrapulmonary manifestations, in this case the AATD-associated liver disease. Development is actually focussing on (1) diversified recombinant protein production platforms for alpha-1-proteinase inhibitor augmentation therapy, and (2) innovative gene therapies, which may, if successfully, encompass a more holistic therapeutical approach.

## Data Availability

The full data set supporting the conclusions of this article is available upon request from Franziska C Trudzinski.

## References

[1] Crystal, R. G. Alpha 1-antitrypsin deficiency, emphysema, and liver disease. Genetic basis and strategies for therapy. *J. Clin. Invest.* **85**, 1343–1352. 10.1172/JCI114578 (1990). 10.1172/JCI114578 PMC296579 2185272

[2] Strnad P, McElvaney NG, Lomas DA (2020). Alpha1-antitrypsin deficiency. N. Engl. J. Med..

[3] Orphanet Report Series, Prevalence and incidence of rare diseases, Bibliographic data, January 2022: available at https://www.orpha.net/orphacom/cahiers/docs/GB/Prevalenceof_rarediseasesby_alphabetical_list.pdf. Accessed 28 April 2022.

[4] Curiel DT, Vogelmeier C, Hubbard RC, Stier LE, Crystal RG (1990). Molecular basis of alpha 1-antitrypsin deficiency and emphysema associated with the alpha 1-antitrypsin Mmineral springs allele. Mol. Cell Biol..

[5] Fraizer GC, Harrold TR, Hofker MH, Cox DW (1989). In-frame single codon deletion in the Mmalton deficiency allele of alpha 1-antitrypsin. Am. J. Hum. Genet..

[6] Kalsheker N, Hayes K, Weidinger S, Graham A (1992). What is Pi (proteinase inhibitor) null or PiQO?: A problem highlighted by the alpha 1 antitrypsin Mheerlen mutation. J. Med. Genet..

[7] Takahashi H (1988). Characterization of the gene and protein of the alpha 1-antitrypsin "deficiency" allele Mprocida. J. Biol. Chem..

[8] Presotto MA (2022). Clinical characterization of a novel alpha1-antitrypsin null variant: PiQ0Heidelberg. Respir. Med. Case Rep..

[9] Weitz, J. I., Silverman, E. K., Thong, B. & Campbell, E. J. Plasma levels of elastase-specific fibrinopeptides correlate with proteinase inhibitor phenotype. Evidence for increased elastase activity in subjects with homozygous and heterozygous deficiency of alpha 1-proteinase inhibitor. *J. Clin. Invest.* **89**, 766–773. 10.1172/JCI115654 (1992). 10.1172/JCI115654 PMC442920 1541671

[10] Geddes DM (1977). alpha 1-antitrypsin phenotypes in fibrosing alveolitis and rheumatoid arthritis. Lancet.

[11] Fortin PR, Fraser RS, Watts CS, Esdaile JM (1991). Alpha-1 antitrypsin deficiency and systemic necrotizing vasculitis. J. Rheumatol..

[12] Gramegna A (2017). Neutrophil elastase in bronchiectasis. Respir. Res..

[13] Wu Y (1994). A lag in intracellular degradation of mutant alpha 1-antitrypsin correlates with the liver disease phenotype in homozygous PiZZ alpha 1-antitrypsin deficiency. Proc. Natl. Acad. Sci. USA.

[14] Fargion S (1981). Alpha 1-antitrypsin in patients with hepatocellular carcinoma and chronic active hepatitis. Clin. Genet..

[15] Haffner ME, Torrent-Farnell J, Maher PD (2008). Does orphan drug legislation really answer the needs of patients?. Lancet.

[16] FDA. Search Orphan Drug Designations and Approvals. Available from: https://www.accessdata.fda.gov/scripts/opdlisting/oopd/listResult.cfm. Accessed 16 July 2021.

[17] Orphanet. About Orphan Drugs. Available from: https://www.orpha.net/consor/cgi-bin/Education_AboutOrphanDrugs.php?lng=EN&stapage=ST_EDUCATION_EDUCATION_ABOUTORPHANDRUGS_SIN. Accessed 04 August 2022.

[18] Orphanet. About Orphan Drugs. Available from: https://www.orpha.net/consor/cgi-bin/Education_AboutOrphanDrugs.php?lng=EN. Accessed 04 August 2022.

[19] Vandenbroucke JP (2007). Strengthening the reporting of observational studies in epidemiology (STROBE): Explanation and elaboration. PLoS Med..

[20] FDA. FDALabel. Available from: https://nctr-crs.fda.gov/fdalabel/ui/search. Accessed 05 August 2021.

[21] Johann P, Lenz D, Ries M (2021). The drug development pipeline for glioblastoma—A cross sectional assessment of the FDA Orphan Drug Product designation database. PLoS ONE.

[22] Garbade SF (2020). FDA orphan drug designations for lysosomal storage disorders—a cross-sectional analysis. PLoS ONE.

[23] Clinical Development Success Rates and Contributing Factors 2011–2020. Available from: https://pharmaintelligence.informa.com/~/media/informa-shop-window/pharma/2021/files/reports/2021-clinical-development-success-rates-2011-2020-v17.pdf. Accessed 04 August 2022.

[24] Chapman KR (2015). Intravenous augmentation treatment and lung density in severe alpha1 antitrypsin deficiency (RAPID): A randomised, double-blind, placebo-controlled trial. Lancet.

[25] McElvaney NG (2017). Long-term efficacy and safety of alpha1 proteinase inhibitor treatment for emphysema caused by severe alpha1 antitrypsin deficiency: An open-label extension trial (RAPID-OLE). Lancet Respir. Med..

[26] Vogelmeier, C. F. *et al.* Global strategy for the diagnosis, management, and prevention of chronic obstructive lung disease 2017 report. GOLD executive summary. *Am. J. Respir. Crit. Care Med.* **195**, 557–582. 10.1164/rccm.201701-0218PP (2017). 10.1164/rccm.201701-0218PP 28128970

[27] Mechler K, Mountford WK, Hoffmann GF, Ries M (2015). Pressure for drug development in lysosomal storage disorders—a quantitative analysis thirty years beyond the US orphan drug act. Orphanet. J. Rare Dis..

[28] Miravitlles, M. *et al.* European Respiratory Society statement: diagnosis and treatment of pulmonary disease in alpha1-antitrypsin deficiency. *Eur Respir J* **50**, 1. 10.1183/13993003.00610-2017 (2017). 10.1183/13993003.00610-2017 29191952

[29] Clark VC (2018). Clinical and histologic features of adults with alpha-1 antitrypsin deficiency in a non-cirrhotic cohort. J. Hepatol..

[30] Hamesch, K. *et al.* Liver fibrosis and metabolic alterations in adults with alpha-1-antitrypsin deficiency caused by the Pi*ZZ mutation. *Gastroenterology* **157**, 705–719. 10.1053/j.gastro.2019.05.013 (2019). 10.1053/j.gastro.2019.05.013 31121167

[31] Brand P (2009). Lung deposition of inhaled alpha1-proteinase inhibitor in cystic fibrosis and alpha1-antitrypsin deficiency. Eur. Respir. J..

[32] Herth FJF (2021). Alpha 1 antitrypsin therapy in patients with alpha 1 antitrypsin deficiency: Perspectives from a Registry Study and practical considerations for self-administration during the COVID-19 pandemic. Int. J. Chron. Obstruct. Pulmon. Dis..

[33] Wooddell, C. I. *et al.* Development of an RNAi therapeutic for alpha-1-antitrypsin liver disease. *JCI Insight* **5**. 10.1172/jci.insight.135348 (2020). 10.1172/jci.insight.135348 PMC7406265 32379724

[34] Turner AM (2018). Hepatic-targeted RNA interference provides robust and persistent knockdown of alpha-1 antitrypsin levels in ZZ patients. J. Hepatol..

[35] Stockklausner, C., Lampert, A., Hoffmann, G. F. & Ries, M. Novel treatments for rare cancers: The U.S. Orphan drug act is delivering-a cross-sectional analysis. *Oncologist* **21**, 487–493. 10.1634/theoncologist.2015-0397 (2016). 10.1634/theoncologist.2015-0397 PMC4828121 27022038

[36] Döring JH, Lampert A, Hoffmann GF, Ries M (2016). Thirty years of orphan drug legislation and the development of drugs to treat rare seizure conditions: A cross sectional analysis. PLoS ONE.

[37] Lutz T, Lampert A, Hoffmann GF, Ries M (2016). Novel treatments for rare rheumatologic disorders: Analysis of the impact of 30 years of the US orphan drug act. Orphanet J. Rare Dis..

[38] Ademuwagun IA, Rotimi SO, Syrbe S, Ajamma YU, Adebiyi E (2021). Voltage gated sodium channel genes in epilepsy: Mutations, functional studies, and treatment dimensions. Front. Neurol..

[39] Dunleavy, K. The top 20 pharma companies by 2021 revenue. Available from: https://www.fiercepharma.com/special-reports/top-20-pharma-companies-2021-revenue. Accessed 04 Aug 2022 (2022).

[40] *FDA. Public Law 97–414, 97th Congress, An Act To amend the Federal Food, Drug, and Cosmetic Act to facilitate the development of drugs for rare diseases and conditions, and for other purposes 1983.* Available from: https://www.fda.gov/media/99546/download. Accessed 11 Feb 2022.

